# Hepatitis B Virus Middle Protein Enhances IL-6 Production via p38 MAPK/NF-κB Pathways in an ER Stress-Dependent Manner

**DOI:** 10.1371/journal.pone.0159089

**Published:** 2016-07-19

**Authors:** Yang-Xia Li, Yan-Li Ren, Hai-Jing Fu, Ling Zou, Ying Yang, Zhi Chen

**Affiliations:** 1 State Key Laboratory for Diagnosis and Treatment of Infectious Diseases, Institute of Infectious Diseases, First Affiliated Hospital, School of Medicine, Zhejiang University, Hangzhou, China; 2 Collaborative Innovation Center for Diagnosis and Treatment of Infectious Diseases, Hangzhou, China; 3 School of Medicine, Zhejiang University, Hangzhou, China; CRCL-INSERM, FRANCE

## Abstract

During hepatitis B virus (HBV) infection, three viral envelope proteins of HBV are overexpressed in the endoplasmic reticulum (ER). The large S protein (LHBs) and truncated middle S protein (MHBs^t^) have been documented to play roles in regulating host gene expression and contribute to hepatic disease development. As a predominant protein at the ultrastructural level in biopsy samples taken from viremic patients, the role of the middle S protein (MHBs) remains to be understood despite its high immunogenicity. When we transfected hepatocytes with an enhanced green fluorescent protein (EGFP)-tagged MHBs expressing plasmid, the results showed that expression of MHBs cause an upregulation of IL-6 at the message RNA and protein levels through activating the p38 mitogen-activated protein kinase (p38 MAPK) and nuclear factor-kappa B (NF-κB) pathways. The use of specific inhibitors of the signaling pathways can diminish this upregulation. The use of BAPTA-AM attenuated the stimulation caused by MHBs. We further found that MHBs accumulated in the endoplasmic reticulum and increased the amount of glucose regulated protein 78 (GRP78/BiP). Our results provide a possibility that MHBs could be involved in liver disease progression.

## Introduction

During hepatitis B virus (HBV) infection, three surface proteins are produced by two promoters, the preS1 and preS2 promoters, in the endoplasmic reticulum (ER) [1]. The preS1 promoter triggers the transcription of the large S protein (LHBs, which contains the preS1, preS2 and S domains), while the preS2 promoter initiates the expression of the middle S protein (MHBs, contains the preS2 and S domains) and the small S protein (SHBs, contains the S domain) [2]. When expressed alone, the MHBs and SHBs proteins can be secreted while the LHBs protein is retained in the ER [3]. The aggregation of LHBs initiates ER stress and plays a potential role in HBV-related hepatocarcinogenesis [4]. The MHBs is unnecessary for virion formation, secretion and infectivity [5], but it is important to induce human immune responses in the early stage of HBV infection [6]. At same time, as the most immunogenic protein among the three surface antigens [7], the antibody against preS2 can inhibit HBV infection [8]. Recently, MHBs was reported to be involved in the regulation of host gene transcription [9] and the synthesis of covalently closed circular DNA (cccDNA) [10]. During chronic HBV infection, the dysregulated overexpression of LHBs cause the retention of MHBs and SHBs in the ER [11], which is related with the progression of hepatitis B [3]. Meanwhile, MHBs was found to be predominant at the ultrastructural level and to exist in the cytoplasm in biopsy samples taken from viremic patients [2].

Interleukin (IL)-6 plays key roles in the development of liver fibrosis and cancers [12–15]. In the early stage of hepatocarcinogenesis, IL-6 is mainly produced by Kupffer cells in a MyD88-TLR dependent manner [16], while in the cancer progression, autocrine IL-6 is produced by hepatic stellate cells (HSCs) [17] and hepatocellular carcinoma (HCC) progenitor cells (HcPCs) through a nuclear factor-kappa B (NF-κB) -Lin28-Let7 pathway [18]. HBV infection also contributes to the enhancement of IL-6 levels in the serum and the liver [19,20], which may partially account for the association of HBV infection and the increased risk of HCC development [21]. In addition, HBV X protein triggers human hepatocytes to secrete IL-6 by activating NF-κB in a MyD88-dependent manner and is regulated by PP2Cα [22–24]. C-terminus truncated MHBs (MHBs^t^) and LHBs can also activate NF-κB [25,26], which is a regulator of IL-6 production.

When fused with an enhanced green fluorescent protein (EGFP), the intracellular accumulation of MHBs can be observed [27]. We expressed MHBs fused with an EGFP at the C-terminal end in hepatic and hepatoma cells. Our results showed that MHBs activated p38 mitogen-activated protein kinase (p38 MAPK) and NF-κB pathways to stimulate the production of IL-6. The use of specific inhibitors of these signaling pathways and ER stress can diminish the MHBs-induced stimulation.

## Materials and Methods

### Cell lines and culture

Human hepatic L-02 cells, hepatoma Huh7 cells and hepatoma SMMC-7721 cells were cultured in DEME (Corning, USA) containing 10% fetal calf serum (Gibco, USA). The Huh7 cells were from the American Type Culture Collection, while L-02 cells and SMMC-7721 cells were obtained from the Cell Bank of Type Culture Collection of Chinese Academy of Science, Shanghai Institute of Cell Biology, Chinese Academy of Science.

### Plasmid construct and transfection

The plasmid MHB-GFP was constructed by cloning the full length of the open reading frame of the HBV middle protein from pcDNA3.0–1.3HBV into the pEGFP-N1 vector using Hind Ⅲ (TAKARA, Japan) and KpnⅠ(TAKARA, Japan) restriction sites. We used the pEGFP-N1 vector that expresses GFP protein as a control. The cyan fluorescent protein (CFP) fused ERD-2-like protein (ELP-1-CFP) encoding plasmid was provided as a gift by professor Wei Liu at Zhejiang University.

Transient transfection was performed using Lipofection 3000 regent (Invitrogen, USA). Briefly, we settled approximately 8×10^5^ cells per well in a 6-well plate overnight and then transfected 2.5 μg of plasmid per well. The cells and culture supernatants were collected to analyze the gene expression at the mRNA and/or protein levels after a 24-hour transfection.

### ELISA analysis

ELISA kits (Lianke, China) were used to detect IL-6 protein levels in cell culture supernatants. Before detection, the supernatants were centrifuged at 1,000 × g for two minutes.

### Real-time RT-PCR analysis

Total RNA was extracted using an EastepTM Universal RNA Extraction Kit (Promega, USA). In total, 2 μg of RNA was reverse transcribed using a PrimeScript™ RT reagent Kit with gDNA Eraser (Perfect Real Time; TAKARA, Japan), and 2 μL of cDNA was amplified with SYBR^®^ Premix Ex Taq™ II (Tli RNaseH Plus; TAKARA, Japan) using an ABI7500 (USA). The primers for IL-6 and β-actin (as an invariant housekeeping gene internal control) were from Xiang *et al*. [24].

### Western blot analysis

Halt protease and phosphatase inhibitor cocktail (Thermo Scientific, USA) were added to RIPA buffer (Millipore, Germany) at a ratio of 5:100; the mixture was used to lyse cells to prepare protein samples. Protein samples were separated in 10% sodium dodecyl sulfate polyacrylamide gels and transferred onto polyvinylidene difluoride (PVDF) membranes (Millipore, Germany). After blocking with 5% bovine serum albumin (BSA), the membranes were incubated with specific primary antibodies (1:1,000) and then HRP-conjugated secondary antibodies (1:5,000). The primary antibodies comprised antibodies against GAPDH (HuaAn Biotechnology, China), preS2 (Abcam, USA), p44/42, phospho-p44/42, p38, phospho-p38, IκB (Cell Signaling, USA) and BiP (Abcam, USA). The specific bands were detected with an EZ-ECL Kit (Biological Industries, Israel). The relative levels of target proteins to GAPDH were determined with ImageJ software.

### Immunofluorescent assay

Approximately 5×10^3^ cells per well were settled in a 24-well plate overnight and then transfected with 1 μg of plasmids per well. After incubation for 24 hours, the cells were fixed with 4% paraformaldehyde, treated with 0.5% Triton-X 100, blocked with 1% BSA at room temperature, and then incubated with specific primary antibodies against p65 (Santa Cruz, USA) or preS2 (Abcam, USA) at 4℃ overnight. Subsequently, the cells were incubated with an Alexa Fluor® 568 conjugated donkey anti-Rabbit IgG (H+L) secondary antibody or Alexa Fluor® 568 conjugate goat anti-Mouse IgG (H+L) secondary antibody (ThermoFisher Scientific, USA) for one hour and then incubated with DAPI for 5 minutes at room temperature. The expression of MHB and the subcellular localization of p65 were observed with a Zeiss confocal microscope (LSM 510 Meta, Germany). For the subcellular localization of MHBs, MHBs and ELP-1-CFP were cotransfected into SMMC-7721 cells and observed with a Nikon confocal microscope (A1R, Japan).

### Effects of inhibitors on IL-6 stimulation

Approximately 5×10^4^ cells per well were settled in a 24-well plate. Twelve hours after transfection, the cells were washed three times; cultured in fresh medium containing 10 μM of QNZ (Selleck, USA), 20 μM of SB203580 (Selleck, USA) or both; and then incubated for another twelve hours. The culture supernatants were collected to determine the IL-6 protein level using ELISA kits (Lianke, China).

Approximately 8×10^5^ cells per well were settled in a 6-well plate. Twenty-two hours after transfection, the cells were washed and cultured in fresh medium containing 10 μM, 20 μM or 40 μM of 1,2-bis (2-aminophenoxy) ethane-N,N,N’,N’-tetraacetic acid (BAPTA-AM) (Selleck, USA) for another 2 hours. The cells were collected to analyze the phosphorylation levels of p38 MAPK and IκB. The culture supernatants were used to detect the IL-6 protein level using ELISA kits (Lianke, China).

### Statistical analysis

Statistical analysis was performed by SPSS (Version 16.0; Chicago, IL). A *p* value<0.05 was considered as statistically significant. One-way ANOVA was used to analyze the data among treatments, the letters above the bars indicate significant differences (*p*<0.05) among treatments. *t-tests* were used to analyze the data between two groups, “*” above the bars indicates *p*<0.05 while “**” means *p*<0.01.

## Results

### MHBs stimulates the expression and secretion of IL-6

**Fig 1:**
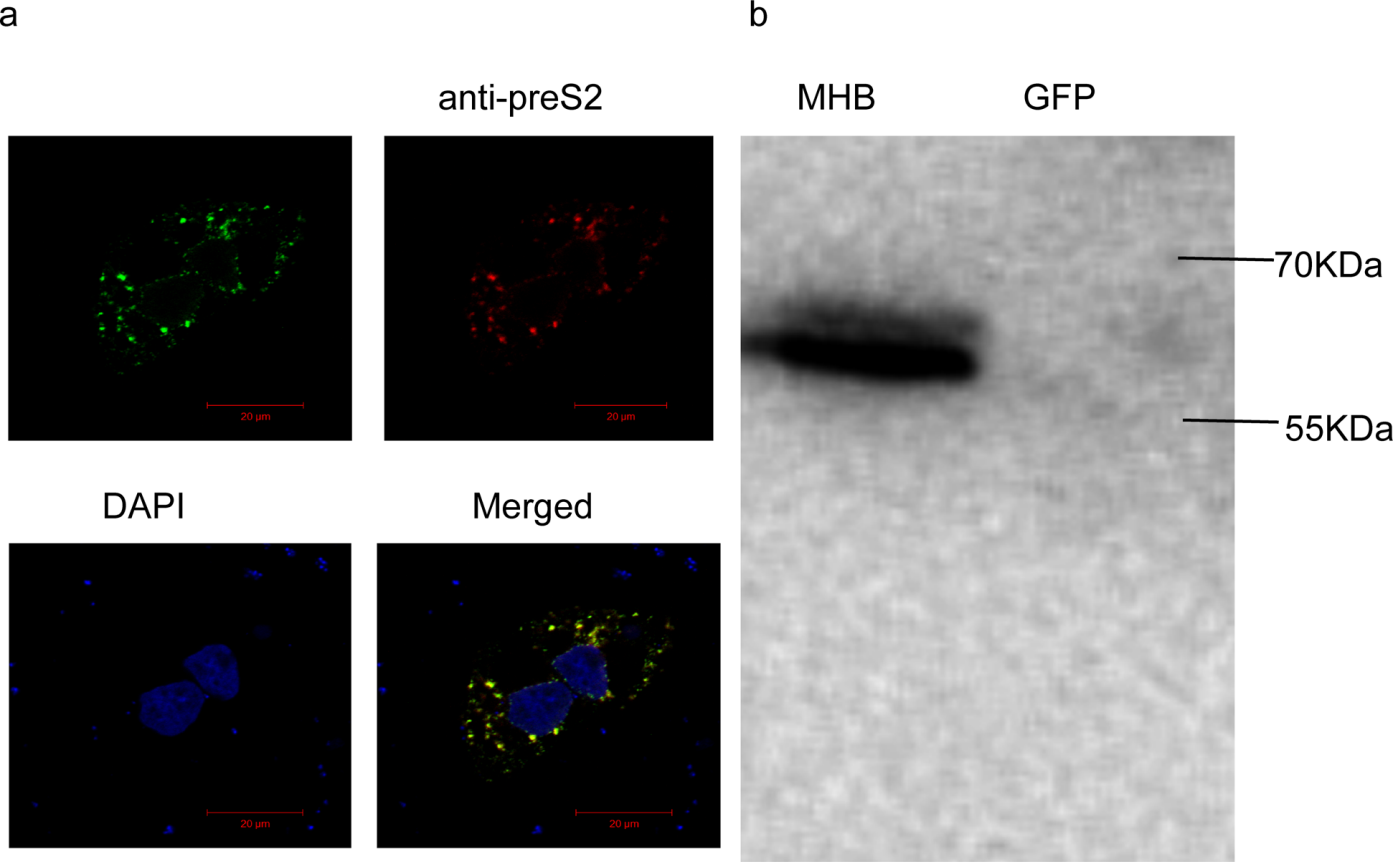
MHBs accumulated in Huh7 cells. (a) The intracellular accumulation of MHBs was determined with anti-preS2 antibody. (b) Proteins were collected from cells at 24 hours after transfection and were analyzed by western blot using an anti-preS2 antibody.

The intracellular accumulation and expression of MHBs was detected with anti-preS2 antibody using immunofluorescent (Fig 1A) and western blot analysis (Fig 1B).

**Fig 2:**
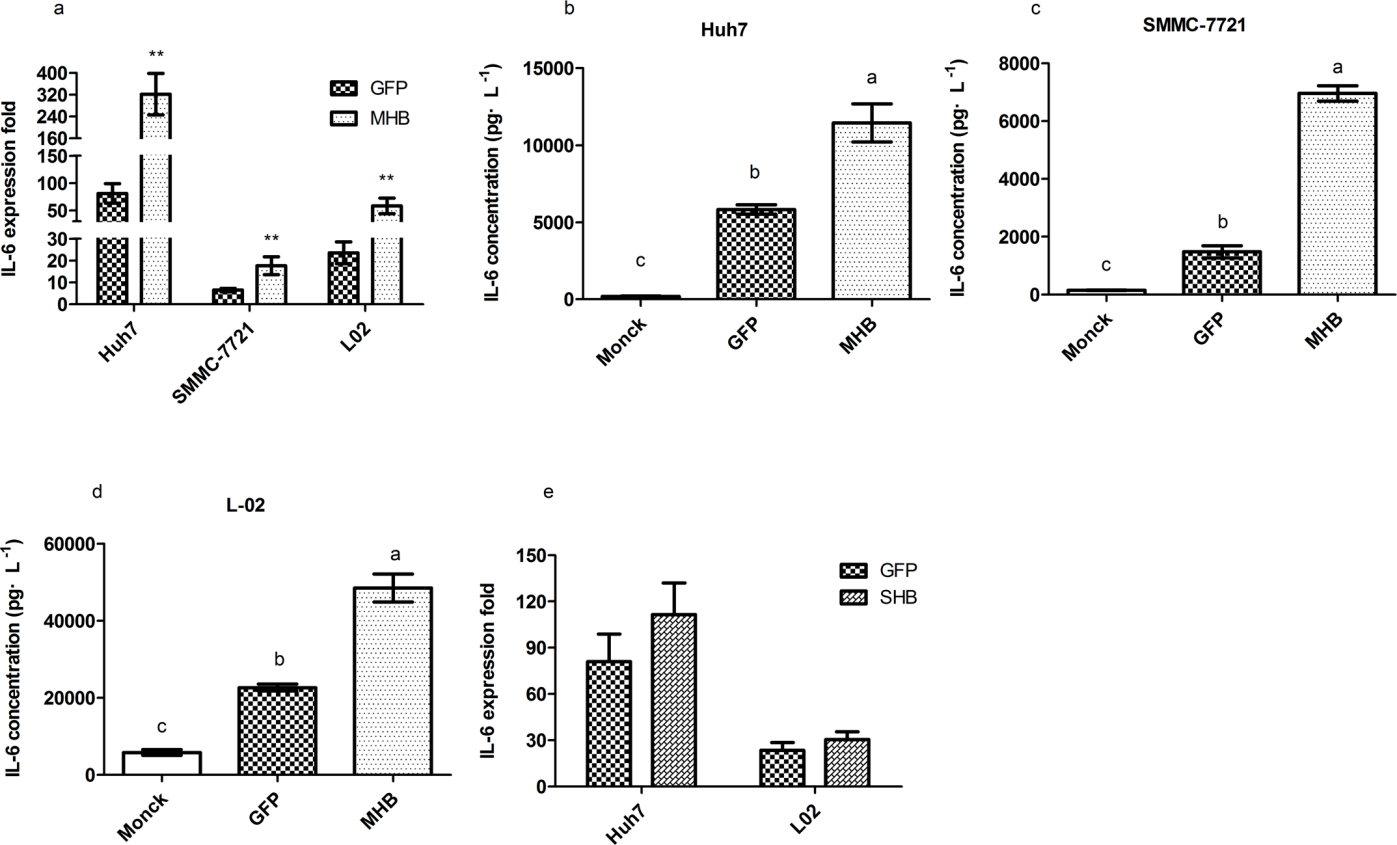
MHBs stimulates IL-6 production in hepatocytes and hepatoma cells. Huh7, SMMC-7721 and L-02 cells were transfected with MHB as well as with GFP plasmid to detect *IL-6* mRNA levels (a) and IL-6 protein levels (b-d).(e) Huh7 and L-02 cells were transfected with SHB as well as with GFP plasmid to detect *IL-6* mRNA levels.

After transfection for 24 hours, the total RNA and cell culture medium from human hepatic L-02 cells, hepatoma SMMC-7721 cells and hepatoma Huh7 cells were collected. The results showed that the expression of MHBs significantly induced the transcription and secretion of IL-6 compared to GFP plasmid transfection (Fig 2A–2D). It has been reported that cells infected with a recombinant adenovirus which contained the middle envelope gene expressed and secreted the middle and small envelope proteins in a molar ratio of 3:1 [28]. To verify the increasing of IL-6 was induced by MHBs, we transfected Huh7 cells and L-02 cells with SHBs-GFP plasmid and detected the levels of *IL6* mRNA. The data showed no significant difference between SHBs and GFP groups (Fig 2E).

### MHBs activates p38 MAPK and NF-κB pathways

**Fig 3:**
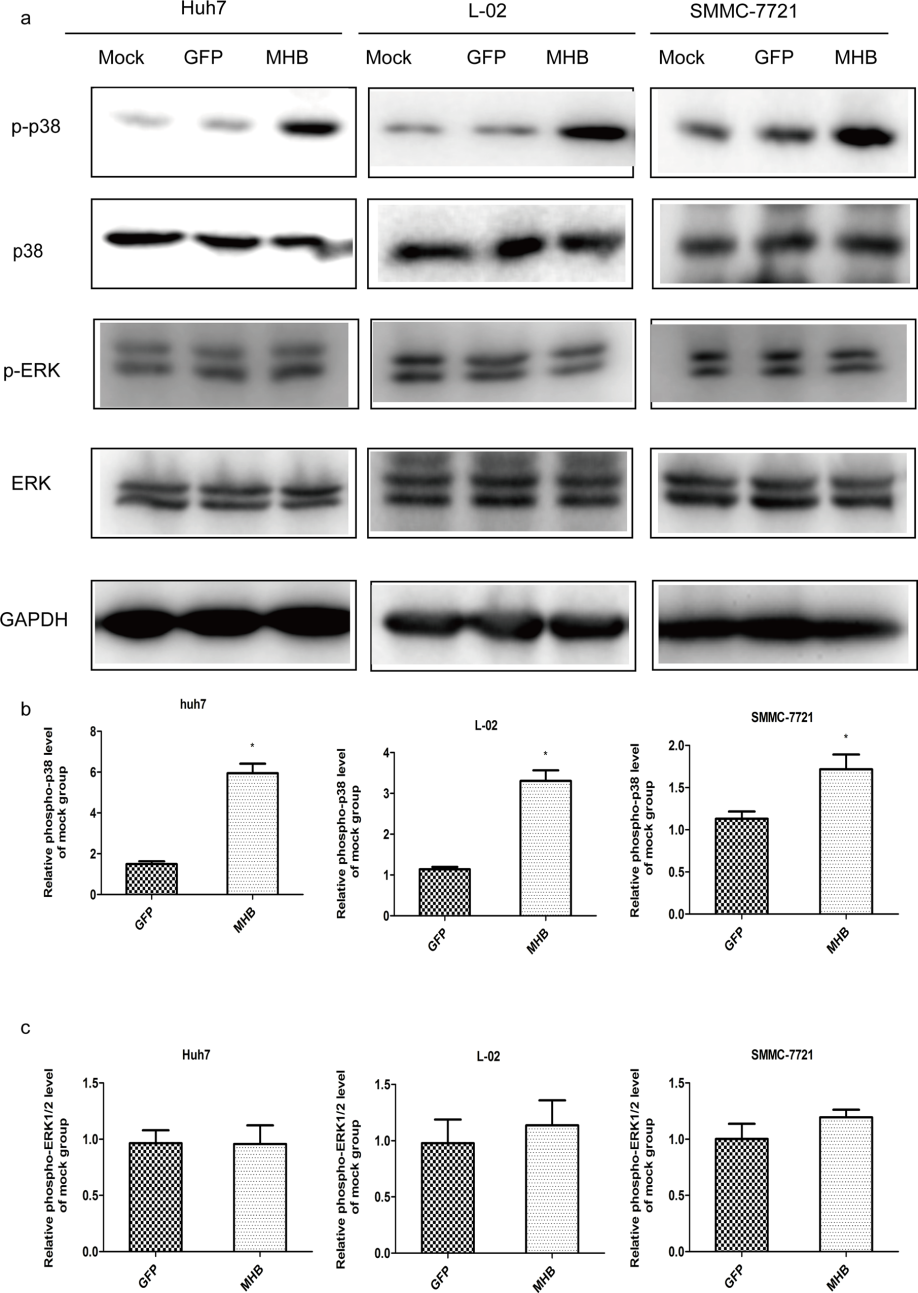
MHBs stimulates the phosphorylation of p38 but not ERK1/2. Huh7, L02 and SMMC-7721 cells were transfected with MHB or GFP. (a) The phosphorylation of p38 and ERK1/2 were determined using specific antibodies and western blot. (b and c) The data from three independent experiments were analyzed with a paired *t-test*.

**Fig 4:**
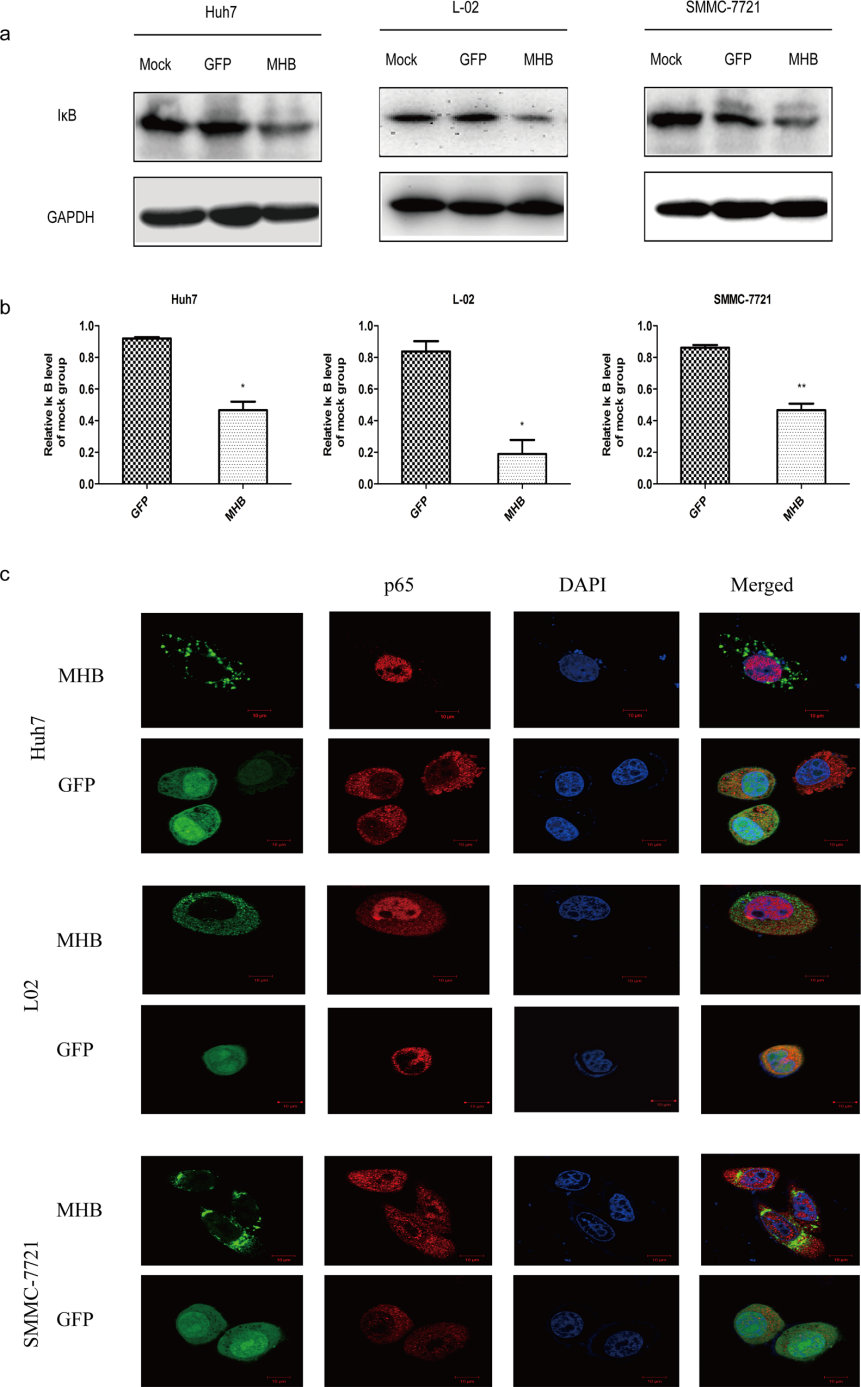
MHBs activates NF-κB. Huh7, L-02 and SMMC-7721 cells were transfected with MHB or GFP. (a) The degradation of Iκ-B was detected by western blot. (b) The data from three independent experiments were analyzed using paired *t-tests*. (c) The nucleus-location of the p65 subunit was determined by immunostainig.

IL-6 production can be regulated by activating protein-1 (AP-1) [29], NF-κB and CCAAT/enhancer-binding protein (C/EBP) [30]; the p38 MAPK and extracellular signal-related kinase (ERK) signal pathways are also involved [24,31,32]. To investigate the signaling pathways and transcriptional factors involved in MHBs-induced IL-6 expression, we checked the activity of MAPK by using specific anti-phospho-p38 antibody and anti-phospho-ERK (Thr202/Tyr204) antibody. Our results showed that MHBs enhanced the phosphorylation of p38 but not ERK1/2 (Fig 3). The activation of NF-κB was determined by the degradation of Iκ-B and the nucleus-translocation of the p65 subunit. Our results showed that MHBs caused the degradation of Iκ-B and nucleus-translocation of the p65 subunit (Fig 4).

### Activation of p38 MAPK/NF-κB pathways is necessary for MHBs-induced IL-6 production

**Fig 5:**
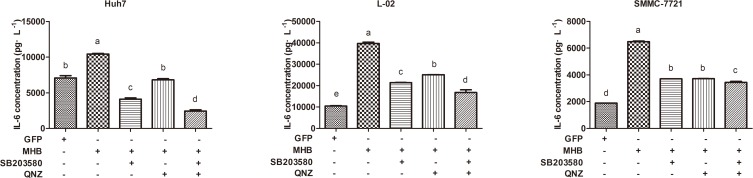
Activation of p38 and NF-κB pathway is necessary for MHBs-induced IL-6 production. Huh7, L-02 and SMMC-7721 cells were transfected with MHB or GFP, cells were treated with SB203580 and QNZ individually or both, the IL-6 protein levels were analyzed.

To confirm the molecular mechanisms of MHBs-induced upregulation of IL-6 expression, the specific inhibitors SB203580 (p38 MAPK inhibitor) and QNZ (NF-κB inhibitor) were employed. After transfection, the cells were pretreated with the two inhibitors for 12 hours, and IL-6 expression was checked. The results showed that the production of IL-6 can be diminished significantly by both inhibitors and especially by the combined use of SB203580 and QNZ (Fig 5).

### ER stress is necessary for the activation of p38/NF-κB pathways and upregulation of IL-6 by MHBs

**Fig 6:**
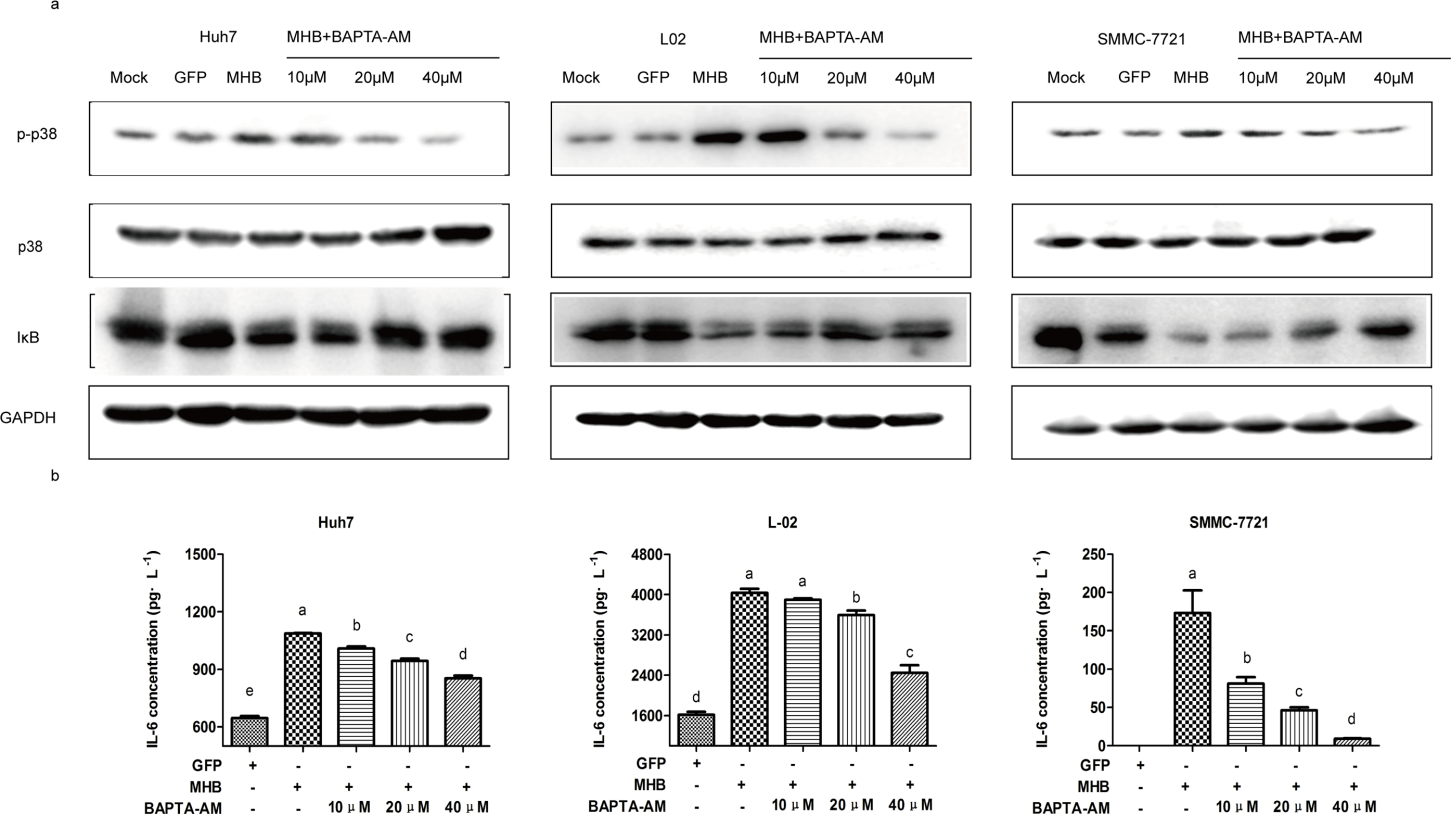
ER stress is necessary for MHBs-induced p38/NF-κB activation and an increase in IL-6. Huh7, L-02 and SMMC-7721 cells were transfected with MHB or GFP, the cells were treated with BAPTA-AM. (a) The phosphorylation of p38 MAPK and degradation of Iκ-B were detected by western blot. (b) The IL-6 protein level in the supernatants was detected by ELISA.

Because it has been reported that the activation of p38 and NF-κB are related to ER stress [26], to investigate whether ER stress was involved in the stimulation caused by MHBs, we pretreated cells with an intracellular calcium chelator, BAPTA-AM. The results showed that the MHBs-stimulated phosphorylation of p38, degradation of Iκ-B and secretion of IL-6 were attenuated by BAPTA-AM in a dose-dependent manner (Fig 6).

### MHBs initiates ER-stress

**Fig 7:**
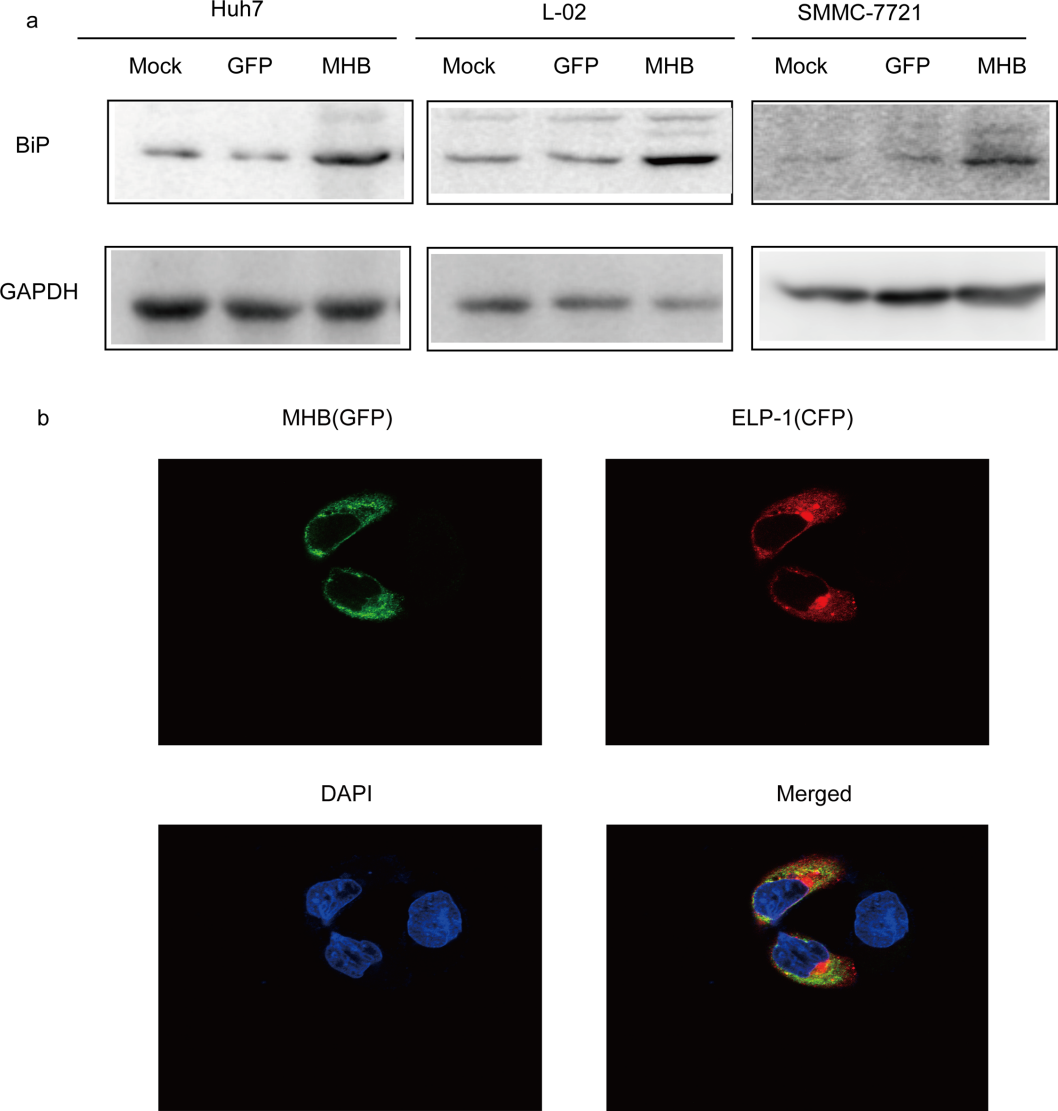
MHBs stimulates ER stress. (a) Huh7, L02 and SMMC-7721 cells were transfected with MHB or GFP. The expression of BiP was determined with a specific antibody and western blot. (b) SMMC-7721 cells were cotransfected with ELP-1-CFP and MHB, the colocalization of the ELP-1 and MHB was observed.

To verify the role of ER stress in MHBs-stimulated IL-6 production, we detected the variation of glucose regulated protein 78 (GRP78/BiP) in the case of MHBs expression. Our results showed that MHBs enhanced the amount of BiP (Fig 7A). We further cotransfected an ER marker (ELP-1-CFP) and MHBs into SMMC-7721 cells; the colocalization of ELP-1 and MHBs was observed (Fig 7B), indicating that MHBs accumulated in the ER.

## Discussion

In this study, we used two hepatoma cell lines and a hepatic cell line to study the effects of MHBs on cellular gene expression. The results indicated that MHBs activated p38 MAPK and NF-κB to increase the production of IL-6. The use of specific inhibitors confirmed the molecular link between p38 MAPK/NF-κB and IL-6. In addition, the employment of BAPTA-AM, BiP expression analysis and the location of MHBs in the ER indicated that the MHBs-induced stimulation was dependent on ER stress.

HBV belongs to the hepadnaviruses family and has approximately 3.2 kb of its genome covered by 4 partially overlapping open reading frames (ORFs), which include the pre-S/S ORF, the pre-C/C ORF, the P ORF and the X region. The three viral envelope proteins, termed LHBs, MHBs and SHBs, are encoded by the preS/S ORF and are translated in the ER of HBV-infected cells. Importantly, the production of these proteins far exceeds that needed for the assembly of virions [5]. Meanwhile, during chronic HBV infection, the integration of viral DNA into the host chromosome may cause viral genomic disruptions and then induce a dysregulation of surface protein production, which in turn leads to the overexpression of LHBs and the accumulation of MHBs and SHBs in the ER [6,11]. The aggregation of mutant HBV surface antigen variants were reported to cause ER stress [4,33]. Persistent ER stress activates autophagy, which is beneficial for hepatocyte survival and viral persistence. Still, viral persistence is a high risk factor for liver cancer [34]. We showed that MHBs-induced activation of p38 and NF-κB was dependent on ER stress.

The secretion rates of MHBs fused with an EGFP at the N-terminus were significantly lower than the wild-type protein [27]. In our study, the coding sequence for MHBs was cloned into a pEGFP-N1 vector, resulting in a recombinant MHBs fused with EGFP at the C-terminus. This action may also induce a slower secretion process and thus cause the cytoplasmic aggregation of MHBs. Recently, a study showed that MHBs display a transactivator function when fused with the ER retention signal ‘KDEL’ at the C-terminal end [9], but the full-length MHBs was thought to have no transactivation function because the orientation of its preS2 domain is oriented toward the lumen [35]. Considering the literature and our data, we propose the possibility that MHBs might not be transactivators but the accumulation of MHBs in the cytoplasm might act as a stimulator.

The literature has suggested that the p38 MAPK and ERK signaling pathways are involved in IL-6 production [24,31,32]. In IL-1β-stimulated IL-6 production, the ERK-pathway appears to be particularly important in normal articular human chondrocytes [31]. However, in retinal Müller cells, p38 MAPK has a stronger regulatory function [32], while the two signaling pathways showed synergistic action in HBx-induced IL-6 expression [24]. Our data showed that in MHBs-induced IL-6 expression, p38 MAPK but not ERK was involved, as the phosphorylation of p38 MAPK was elevated significantly (Fig 2) and the production of IL-6 diminished significantly by SB203580 (Fig 4) while there was no significant difference in phosphorylation of ERK between MHBs-GFP and GFP groups (Fig 2). The ERK and p38 MAPK are two distinct pathways, although there is “cross-talk” between them. The ERK pathway primarily mediates cellular responses to growth factors, while the p38 MAPK pathway primarily mediates cellular responses to stresses [36]. The MHBs accumulation has no influence on ERK but can activate the p38 MAPK pathway, which suggests that MHBs accumulation primarily acts as a stress inducer in hepatocytes.

Meanwhile, L-02 cells secrete more IL-6 than hepatoma cells, and the same result was also observed in HBx-induced IL-6 production [24]. IL-6, as well as HBV envelope-specific cytotoxic T lymphocytes (CTL), helps to eliminate viruses by repressing HBV replication and gene transcription in hepatocytes at the early stage of virus infection [6,37]. Still, this repression might cause a weak immune response which contributes to viral persistence and chronic infection because the reduced expression of viral antigens is not sufficient for immune recognition [6]. In hepatoma cells, IL-6-induced signal transducer and activator of transcription 3 (STAT3) activation facilitates viral gene transcription [38] and is required for HCC formation and growth [39]. These investigations indicated that through IL-6, MHBs have different functions at different HBV infection stages and might play complex roles in liver disease pathogenesis.

The present study identifies intracellular accumulation of MHBs as a transcription activator to stimulate hepatic and hepatoma cells to produce IL-6 through p38 MAPK/NF-κB pathways in an ER stress dependent manner. These data thus provide evidence for the potential that MHBs, in addition to other viral proteins, contribute to liver disease progression during HBV infection.
